# A preference to look closer to the eyes is associated with a position-invariant face neural code

**DOI:** 10.3758/s13423-023-02412-0

**Published:** 2023-11-06

**Authors:** Puneeth N. Chakravarthula, Miguel P. Eckstein

**Affiliations:** 1 Psychological and Brain Science, University of California, Santa Barbara, CA USA; 2 Department of Radiology, Washington University in St. Louis, 4525 Scott Ave, St. Louis, MO 2126 B63110 USA

**Keywords:** Face perception, Eye movements, Adaptation aftereffects, Individual differences

## Abstract

When looking at faces, humans invariably move their eyes to a consistent preferred first fixation location on the face. While most people have the preferred fixation location just below the eyes, a minority have it between the nose-tip and mouth. Not much is known about whether these long-term differences in the preferred fixation location are associated with distinct neural representations of faces. To study this, we used a gaze-contingent face adaptation aftereffect paradigm to test in two groups of observers, one with their mean preferred fixation location closer to the eyes (upper lookers) and the other closer to the mouth (lower lookers). In this task, participants were required to maintain their gaze at either their *own* group’s mean preferred fixation location or that of the *other* group during adaptation and testing. The two possible fixation locations were 3.6° apart on the face. We measured the face adaptation aftereffects when the adaptation and testing happened while participants maintained fixation at either the *same* or *different* locations on the face. Both groups showed equally strong adaptation effects when the adaptation and testing happened at the *same* fixation location. Crucially, only the upper lookers showed a partial transfer of the FAE across the two fixation locations, when adaptation occurred at the eyes. Lower lookers showed no spatial transfer of the FAE irrespective of the adaptation position. Given the classic finding that neural tuning is increasingly position invariant as one moves higher in the visual hierarchy, this result suggests that differences in the preferred fixation location are associated with distinct neural representations of faces.

## Introduction

Humans land their first eye movement on the face at a consistent location, typically at a featureless point below the eyes (Peterson & Eckstein, 2012). This location, referred to as the *preferred fixation location* (PFL), has a functional role in various face tasks like identity, gender, and emotion recognition. When observers are forced to maintain fixation at various locations along the vertical midline of the face, they perform the best at their PFL on these tasks (Peterson & Eckstein, 2012), suggesting a functional role of the PFL in face processing. The variation of performance with fixation location on the face has been captured by a computational model known as the Foveated Ideal Observer (FIO), which performs optimally given the distribution of task-relevant information on the face and the constraints of a foveated visual system (Or et al., 2015; Peterson & Eckstein, 2012). Thus, the FIO shows that the region below the eyes is the optimal region on the face to fixate for maximizing performance on various face-identification tasks.

While most individuals (~ 90%) have their PFL just below the eyes, the PFL also varies moderately across individuals (Peterson & Eckstein, 2013). About 10% of the population has a PFL closer to the mouth (Chakravarthula & Eckstein, 2022). These individual differences generalize to persons embedded in scene images (Broda & Haas, 2022a), to videos containing persons (Broda & de Haas, 2022b), and even to real-world scenarios, as shown by recent experiments measuring gaze position with a portable eye tracker (Peterson et al., 2016). Why do these differences exist? What are the perceptual and neural consequences of these differences? Given the complex and intertwined nature of eye movement and face-feature learning through an individual's life, these are challenging questions to answer. However, research into these questions is important, as several studies have found an increased tendency toward fixating on the mouth in various clinical conditions, such as autism (Tanaka & Sung, 2016), acquired prosopagnosia (Orban de Xivry et al., 2008), bilateral amygdala damage (Adolphs et al., 2005), fragile X syndrome (Hong et al., 2019), and 22q11.2 deletion syndrome (Campbell et al., 2010). Further, normal aging was also associated with increased lower fixations in emotion recognition tasks (Circelli et al., 2013; Sullivan et al., 2007).

One possibility could be that the differences in fixation preference are idiosyncratic and unlikely to affect the high-level cognitive processing of faces. However, several lines of recent research have revealed that the neural representations of high-level stimulus categories can be retinotopic or spatially specific, and are shaped by perception-action loops (Groen et al., 2022). For example, Golomb & Kanwisher (2012) showed that higher visual regions code information in the retinotopic frame and suggest that subjective percept of invariance of visual information with spatial position is constructed dynamically by combining the incoming features with eye position signals. Further, there is increasing evidence that the neural representations of faces are tuned to the typical fixation position. In other words, the neural responses are maximized when the face features fall at typical locations relative to the fixation position (Benjamin de Haas et al., 2016; Issa & DiCarlo, 2012; Stacchi et al., 2019). Therefore, the neural representations of faces might be possibly influenced by the preferred fixation location on the face. In this study, our goal was to explore and characterize this influence using the face adaptation aftereffect (FAE) paradigm.

The FAE is a well-known perceptual effect where prolonged viewing of a face can change the percept of a subsequently viewed face (Webster et al., 2004). The FAE is thought to occur due to visual adaptation (Webster & Macleod, 2011), wherein neurons that code for the features of a stimulus become desensitized after prolonged exposure to the stimulus (Barlow, 1990; but see Solomon & Kohn, 2014). Visual adaptation occurs throughout the visual hierarchy, from the retina to the extrastriate cortex (Webster, 2015). Further, the neurons become more tolerant to changes in low-level features such as retinal position as we move up the visual hierarchy. Given that adaptation aftereffects occur in a stimulus-selective manner, researchers often use the degree of tolerance of these effects to low-level image feature manipulations to infer the cortical locus of perceptual effects (Kohn, 2007; Larsson & Harrison, 2015; Zimmer & Kovács, 2011). For example, Kohn & Movshon (2003) found that contrast adaptation is spatially specific, and inferred that it must occur during the early stages of visual processing. Likewise, the FAE is tolerant to variations in size (Zhao & Chubb, 2001), orientation (Watson & Clifford, 2003), color, and contrast (Yamashita et al., 2005), suggesting the involvement of (relatively) higher-level processing in face perception. Thus, studying the specificity of adaptation aftereffects can provide valuable clues to help us understand neural representations of stimuli.

To characterize the differences in neural representations associated with individual differences in the PFL on the face, we designed a gaze-contingent FAE experimental paradigm to compare the spatial tolerance of the FAE in two groups of individuals who differed in their PFL on the face. A free-eye-movements face-identification task was used to measure the PFLs of a large group of individuals. From this sample, we selected a group of individuals with PFLs high up on the face near the eye region (upper lookers) and another group of individuals with PFLs lower on the face near the mouth region (lower lookers). These groups participated in the gaze-contingent experiment where the strength of their FAE was measured in conditions where the adaptation and testing occurred at the *same* or *different* spatial locations. To match the spatial offsets for testing the position specificity, we selected the *same* and *different* spatial locations to be each group's mean PFL and that of the *other group*, respectively. The finding that the FAE is restricted to the condition where the adapter and test are at the same spatial location would be consistent with the position-specificity of the FAE. Likewise, finding a significant FAE in the condition where the adapter and test are at different spatial locations is consistent with the position-invariance of the FAE.

Our experimental design allows us to measure the variation in position-specificity of the FAE with two independent factors: (a) the PFL on the face and (b) the fixation position relative to the PFL during face adaptation. Since position-specificity is related to the depth of face processing along the visual hierarchy, we can indirectly probe whether the neural representations of faces are modulated by differences in the PFL on the face and the fixation position on the face.

The study was not pre-registered. The de-identified data, stimuli, and analysis scripts to reproduce the findings reported in this paper have been made publicly available via the Open Science Framework (OSF) and can be accessed at https://osf.io/t795b.

## Method

### Overview

The study consisted of two phases. First, we measured the preferred first fixation locations (PFLs) of a large sample of observers. This was done using a free-eye-movements face-identification task. From the large sample of PFLs obtained, we invited back observers whose PFLs were either high up on the face (near the eyes) or low on the face (near the mouth) to participate in the main study. These observers first completed four additional blocks of a free-eye-movements face-identification task. This was done to verify that they consistently maintained their PFL across a larger number of trials. For this part, we used a different set of four faces compared to the prescreening task (see the *Stimuli* section below). After this, they participated in a gaze-contingent face-matching task. This task measured the face-adaptation aftereffect in conditions where adaptation and testing occurred at the same or different fixation locations. The fixation locations were either the observer's *own group* mean-PFL or that of the *other group*. All manipulations were made by moving the stimulus position relative to the fixation location, which was fixed at the center of the screen throughout each trial.

### Experiment design

We used two kinds of experimental paradigms in this paper: the free-fixation face-identification task and the enforced fixation face-matching task to measure the Face Aftereffect (FAE). Below, we describe both tasks in detail.

#### Free fixation face-identification task paradigm

This experimental paradigm was identical to the one used in Chakravarthula & Eckstein (2022). Observers participated in a one-in-five match-to-sample task with faces, where they initiated trials by fixating at one of eight possible peripheral locations. A face randomly selected from the set of five possible faces would flash in the center of the screen, and observers were instructed to move their eyes freely to study the face. After viewing the face for a second, they were required to choose which face was studied. We measured the landing position of the first eye movement onto the face on each trial. These were averaged across 80 trials to calculate the PFL. The free-fixation face-identification paradigm was used twice: first for prescreening and then again to verify the stability of the PFL of the selected individuals. The gap between the pre-screening and the verification study varied from immediately after the prescreening up to 2 weeks after the prescreening. During the verification, we used a one-in-four match-to-sample task (instead of a one-in-five match-to-sample task) with a different set of faces to ensure its generalization across stimuli sets. See the *stimuli* section for the details of the stimuli used in this task.

#### Enforced-fixation face adaptation aftereffects task paradigm

Observers were presented with a face that they had to match to one of two faces (A or B). The test face was sampled from one of the eight possible morphs of faces A or B. During this task observers maintained their gaze at a specific location on the face (which was manipulated). The two fixation locations were chosen on the vertical midline of the face, corresponding to the average PFLs of the two groups (upper and lower lookers). Each observer completed 3 (adapter conditions) × 2 (test face conditions) × 8 (morph levels) × 48 (repeats) = 2,304 trials. The trials were distributed across 24 evenly sized blocks. The three adapter conditions were: no adapter, adapter at the own group's mean PFL, and adapter at the *other group's* average PFL. Likewise, the test face could be shown at the average PFL of the observer's *own group* or that of the *other group*. The fixation positions on the adapter and the test face were fixed within a block, while the test face was chosen uniformly from one of the eight possible morphs.

### Participants

#### Pre-screening

A total of 480 observers participated in a short free-eye-movement face-identification screening task. These participants were undergraduate or graduate students at the University of California, Santa Barbara, and participated in the study either for course credit or a small monetary reward.

#### Face adaptation aftereffects study

Upper and lower lookers were selected based on the measured PFL from the prescreening task. We invited 3.1% of the observers with PFLs furthest up (near the eye region) and down (near the mouth region) along the face from the pool of prescreening task participants for the adaptation tasks. Of these, 14 upper and 11 lower lookers completed the adaptation tasks. We used strict thresholds for the definition of upper and lower lookers. If an observer who qualified in the prescreening failed to maintain their PFL within the required range for upper or lower lookers in the free-fixation face-identification blocks, the observer was excluded from the study. One upper looker and two lower lookers did not complete the adaptation task due to difficulty maintaining fixation for prolonged periods in the adaptation tasks. The upper looker group consisted of three males and 11 females, while the lower looker group consisted of four males and seven females. All participants were students at the University of California, aged 18–25 years. They participated in the experiments in exchange for hourly monetary compensation for 8–12 h. All participants had normal or corrected-to-normal vision.

### Stimuli

#### Prescreening

The prescreening task used the same stimuli as in the prescreening task described in Chakravarthula & Eckstein (2022). The stimulus set consisted of five frontally photographed faces taken from an in-house dataset. A mask was applied so that only the internal features of the faces were visible during the task. The visible portion of the faces was 12.2° in height and 9.9° wide.

#### Face adaptation aftereffects study

**Fig. 1:**
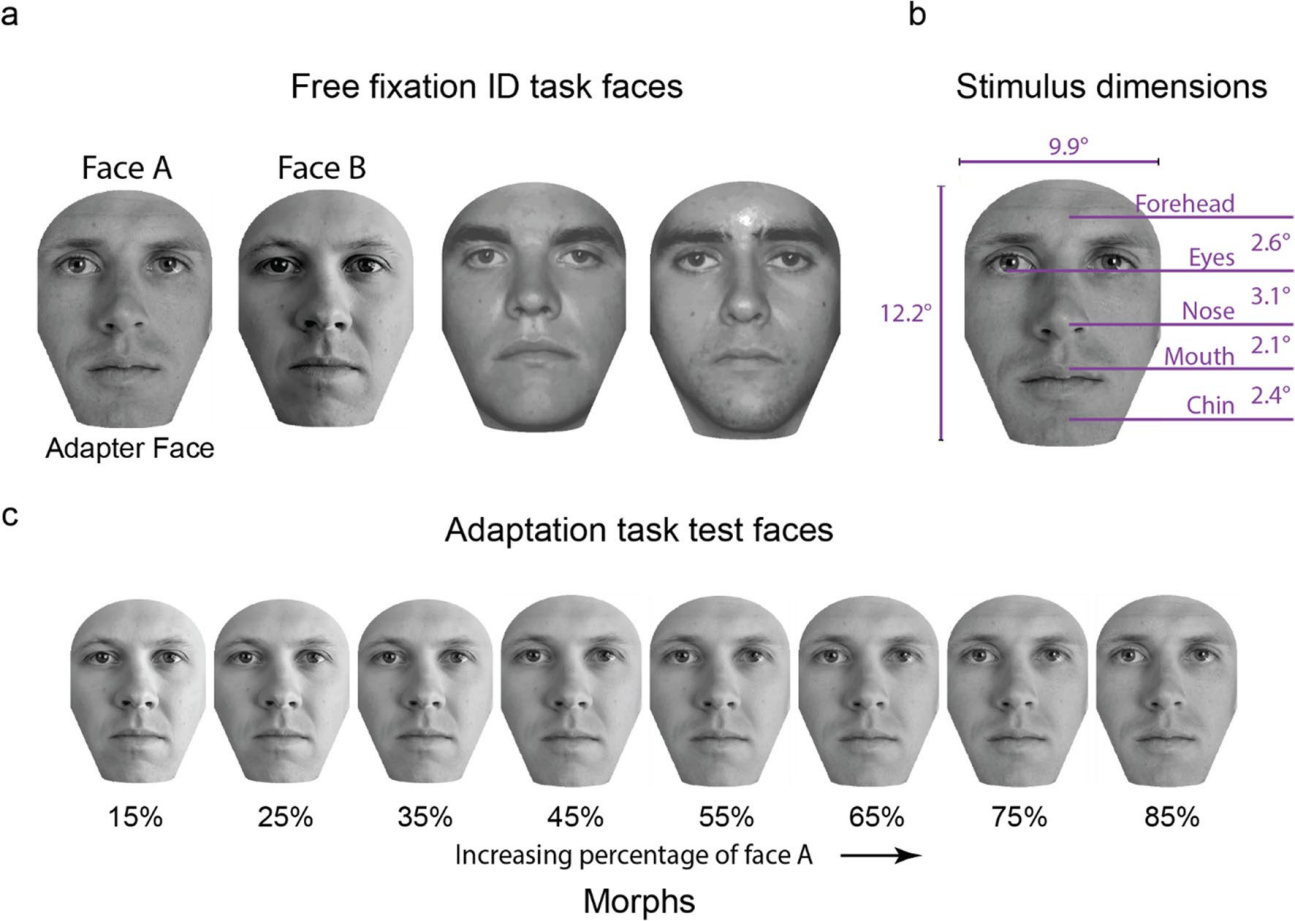
(**a**) The faces used in the free fixation face ID task. Two faces (labeled as faces A and B) were used in the subsequent enforced fixation face-matching task. Only face A was used as the adapter face in the face-matching task. (**b**) The dimensions of the faces used in these tasks. (**c**) The test stimuli used for the enforced fixation face-matching task. From left to right, the faces are created by blending an increasing percentage of face A with a reducing percentage of face B. We used eight different morph levels

The face adaptation aftereffect tasks consisted of two parts. The first part was the free eye movements face-identification task. We used four faces from an in-house face dataset of frontally photographed faces (see Fig. 1a). These faces were preprocessed by rotating, cropping, resizing, and contrast normalization to align and match facial features precisely. A mask was used to cover up all external features, i.e., the hairline and the ears. The faces were 12.2° in height and 9.9° wide (see Fig. 1b). This size falls within 7–14°, the typical range of sizes of male faces encountered during close interpersonal contact (Yang et al., 2014).

Two of the four faces (say A and B, see Fig. 1a) used in the face-identification task were chosen for the subsequent enforced-fixation face-matching task. We created eight morphs using these faces such that the morphs contained 15%, 25%, 35%, 45%, 55%, 65%, 75%, and 85% of face A blended with face B (see Fig. 1c). To do this, we first used a state-of-the-art deep learning-based face landmark registration algorithm to fit 60 landmarks to the two faces, outlining the various features (Bulat & Tzimiropoulos, 2017). The images were then divided into triangles using Delaunay Triangulation. A parametrized affine transform was applied to each of these triangles to generate morphs with the required levels of A and B. Face A was used as the adapter, while the various morphs were used as test faces.

### Apparatus

We used a Barco monitor with a refresh rate of 60 Hz that was linearly calibrated with a maximum luminance of 114.7 cd/m^2^ to display the stimuli. Subjects were seated in a dark room with their eyes located 75 cm from the monitor. An EyeLink 1000 tower-mounted eye tracker was used to track the left eye of each participant. The eye tracker was calibrated with a standard 9-point calibration procedure from EyeLink. The eye-tracker sampling rate was set to 250 Hz. We used a velocity threshold of 30°/s and an acceleration threshold of 9,500°/s^2^ to detect saccades. The stimulus display, eye-tracker data acquisition, and saving were controlled by software (Brainard, 1997) developed in-house and running on MATLAB 2018.

### Trial design

**Fig. 2:**
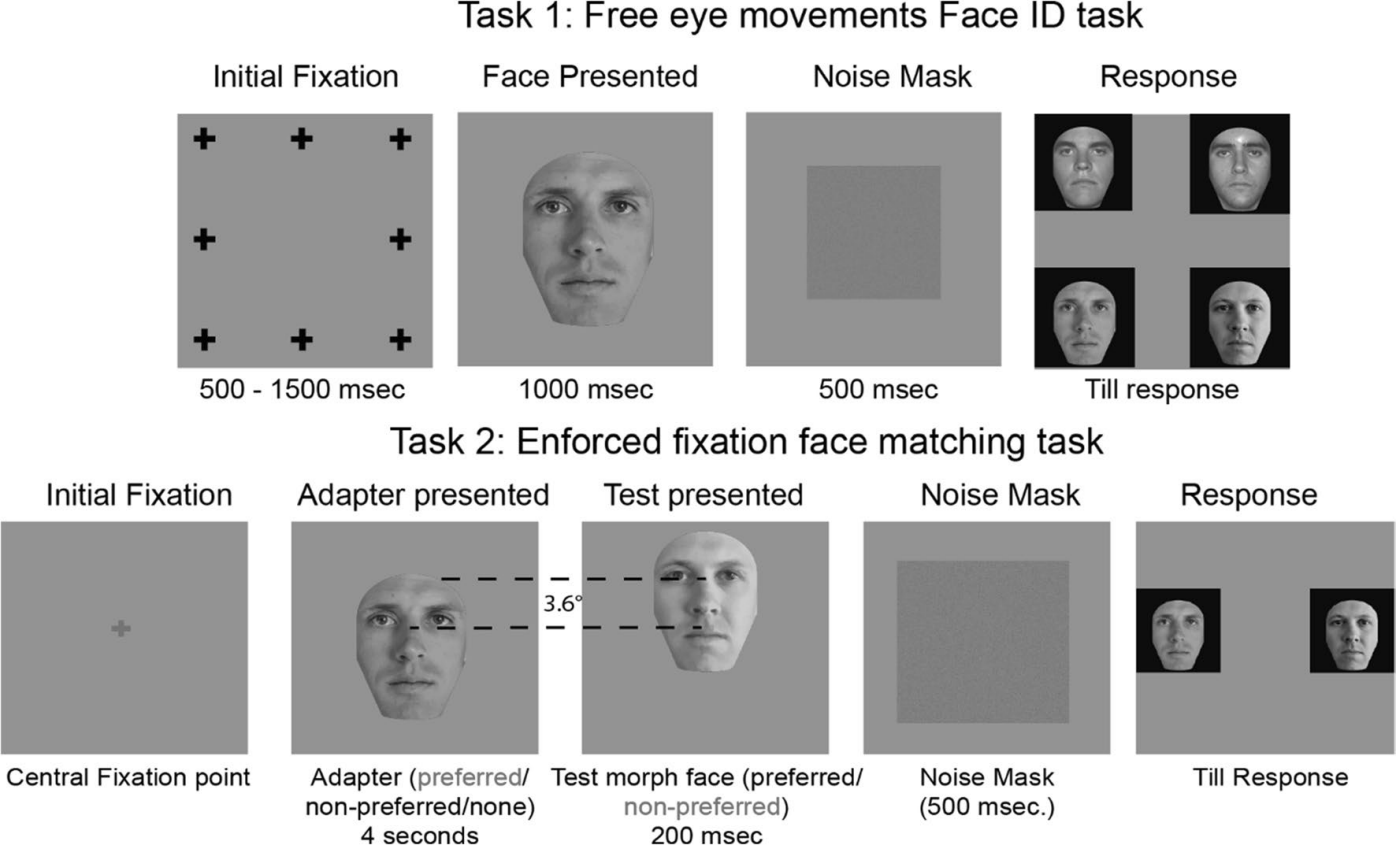
The top panel shows a schematic of the free fixation face identification task. This task was used to assess the landing position of the first eye movement to faces. Observers initiated a trial by fixating on one of eight possible peripheral locations. A face then appeared in the center of the screen, and the observers were instructed to move their eyes freely to study the face. They then had to select which one of the four possible faces was presented. the bottom panel shows a schematic of the forced fixation face-identification task. Observers initiated a trial by fixating the center of the screen. In one-third of the blocks, a test face was directly displayed for 200 ms, such that the observer's fixation lay on the observer's *own group* mean preferred fixation location (*own PFL*), or that of the other group (*other PFL*). In another one-third of the blocks, an adapter first appeared in the preferred position for 4 s. This was followed by a test face in either the observer's *own group PFL* or *other group PFL*. Finally, in another third of the blocks, the adapter was in the other PFL, and the test appeared in either the observer's *own group PFL* or *other group PFL*. After the test face, a response screen was shown with faces A and B. There were 24 blocks, and the sequence of the blocks was randomized for each observer

In this task, the initial fixation always occurred at the center of the screen. The fixation cross was black and overlaid on a gray background (luminance ~ 57 cd/m^2^), which remained unchanged throughout the block. At the beginning of the trial, the observer maintained their gaze at the fixation cross and indicated readiness by a key-press. After the key-press, the program verified if the observer's gaze stayed within 1° of the fixation cross for a variable delay period of 500–1,500 ms. The trial was aborted if the gaze drifted beyond the 1° threshold. The trial progressed if the observer maintained their gaze through the delay period. There were 3 (no adapter/ adapter at PFL/adapter at other groups PFL) × 2 (test at PFL/test at other group's PFL) = six unique conditions in this experiment. Within a block, the observer would only see one condition. If the trial had an adapter, an adapter face was displayed on the screen. The adapter's position was adjusted such that the observer's fixation (which they maintained at the center of the screen) fell on the average PFL of their *own group* or that of the *other group*, based on the condition. The adapter was presented at full contrast for 4 s. The fixation cross was lightened and overlaid on the face as a reference for the observer. During the 2 s, the program checked the observer's gaze to prevent eye movements. If the gaze position drifted beyond 1° from the fixation cross, the trial was terminated. After adaptation, a test face uniformly sampled from the eight morphed faces was flashed for 200 ms. The position of the test face was also adjusted based on the condition (test face at *own group's* average PFL or *the other group's* average PFL). After that, a white Gaussian mask with the mean luminance matched to the background and a standard deviation of 11.2 cd/m^2^ was flashed for 500 ms. The purpose of the mask was to wash out any lingering percept of the test face. Then a response screen with faces A and B (see Fig. 1a) as choices appeared. The screen stayed on until the observer indicated with a mouse press which test face was previously presented. After the response, no feedback was given, and the subsequent trial was initiated. See the lower panel of Fig. 2b for the trial schematic.

### Procedure

The experiments were administered by trained graduate or undergraduate researchers in accordance with protocols approved by the Institutional Review Board (IRB) of The University of California, Santa Barbara. Participants were first briefed about the nature of the study and compensation agreements. After obtaining consent to be a part of the study, they were given instructions about the task. The two tasks (free-fixation identification task and enforced-fixation face adaptation aftereffects task) were always performed in the same order, i.e., the free-fixation task followed by the enforced-fixation task. This was to verify that the participant had a consistent PFL on faces used in the adaptation experiment that matched their PFL as measured in the prescreening task. Note that these tasks were conducted in parallel across observers – as soon as a participant finished the free-fixation task, they could start with the forced-fixation task. This timeline was separate for each participant. Each task was further divided into blocks that took 15–30 min to complete. The free-fixation face-identification task and the enforced-fixation face adaptation aftereffects task had four and 24 blocks, respectively. Participants completed the study in eight to ten sessions spread across 2–4 weeks. They were encouraged to take breaks between blocks and were not allowed to spend more than 1.5 h per session to avoid the effects of fatigue. We recalibrated the eye tracker between blocks and whenever a participant took a break to maintain eye-tracking quality throughout each session.

### Analysis

#### Preferred fixation location

The preferred first fixation location is the first location inside the face that an observer's foveal region lands on when they make an eye movement to a face from a peripheral fixation location. Following the completion of all blocks of the free-fixation task for each observer, the preferred first-fixation location was estimated as the mean fixation location across all the first fixations on the face across trials. The distribution of the coordinates of the first fixations and unimodal and using the mean or median resulted in a similar estimate of the PFL. The vertical coordinates of the first fixation location on the face were used to categorize observers into upper or lower lookers.

#### Strength of adaptation

We first calculated the fraction of times the observer responded to each morph level as face B. We had 48 responses for each morph level in each condition. Thus, the smallest difference in response rate we could measure was ~ 2%. For each condition, we fit a psychometric function of the form

1 $$\psi (x;\gamma ,\lambda ,\mu ,\sigma )=\gamma +(1-\gamma -\lambda )F(x;\mu ,\sigma )$$

where *x* is the % morph level, $\gamma$ is the guess rate, $\lambda$ is the lapse rate, *F* is the cumulative Gaussian function, $\mu$ and $\sigma$ are mean and standard deviations parameters of the Gaussian distribution (Wichmann & Hill, 2001). The accuracy of the fitting was ensured by using the least absolute residual (LAR) method and further by manually examining the fits.

The *point of subjective equality* (PSE), defined as the strength of the stimulus that elicits both possible responses (Face A vs. Face B) with equal probability, was computed from the fit equation for each condition. The strength of adaptation was then calculated as the difference between PSE with adaptation and the PSE with no adaptation, all else being equal.

#### Justification of sample size

The sample size is primarily constrained by the difficulty of finding participants suitable for the study. We chose to test two groups of observers whose PFLs on the face differed by an average of 3.6°. We chose this distance to maximize the chances of finding a difference in the position specificity between the groups while also bearing in mind the prescreening requirements to find the required groups of observers. Before the experiment, we tested a random sample of 15 participants in a pilot experiment to verify if they showed position-invariance in their FAEs. These individuals had their PFLs below the eyes, on the nose. In this pilot sample, there was a significant transfer of the FAE when the adaptation and testing happened 3.6° apart (*t* (14) = 2.62, *p* = 0.02, one-sample *t-*test). Using simulations from an earlier paper (Chakravarthula & Eckstein, 2022), we determined that the number of participants needed to be screened for 20 upper and 20 lower lookers with PFLs differing on an average by 3.6° with an 80% probability is over 1,400 participants. Since we used a stronger PFL manipulation of 3.6° compared to 2.1° in Chakravarthula & Eckstein (2022), we anticipated a greater effect size and needed a smaller sample size to confirm the effects of PFL manipulation. The final tally of 14 upper lookers and 11 lower lookers was thus based on prescreening 480 individuals and selecting participants from the tails of the distributions of the PFLs of these individuals.

#### Outlier identification for robustness

To ensure that our results were robust, we repeated the analyses by removing observers who showed extremely different adaptation strengths for any condition. For this, we identified cases where the adaptation strength deviated from the third quartile of the group by more than 1.5 times the interquartile range (IQR). Only one such case was found, where an upper looker showed a high adaptation strength when they were adapted at the *other group* PFL and tested at their *own group* PFL.

## Results

### Prescreening task

**Fig. 3:**
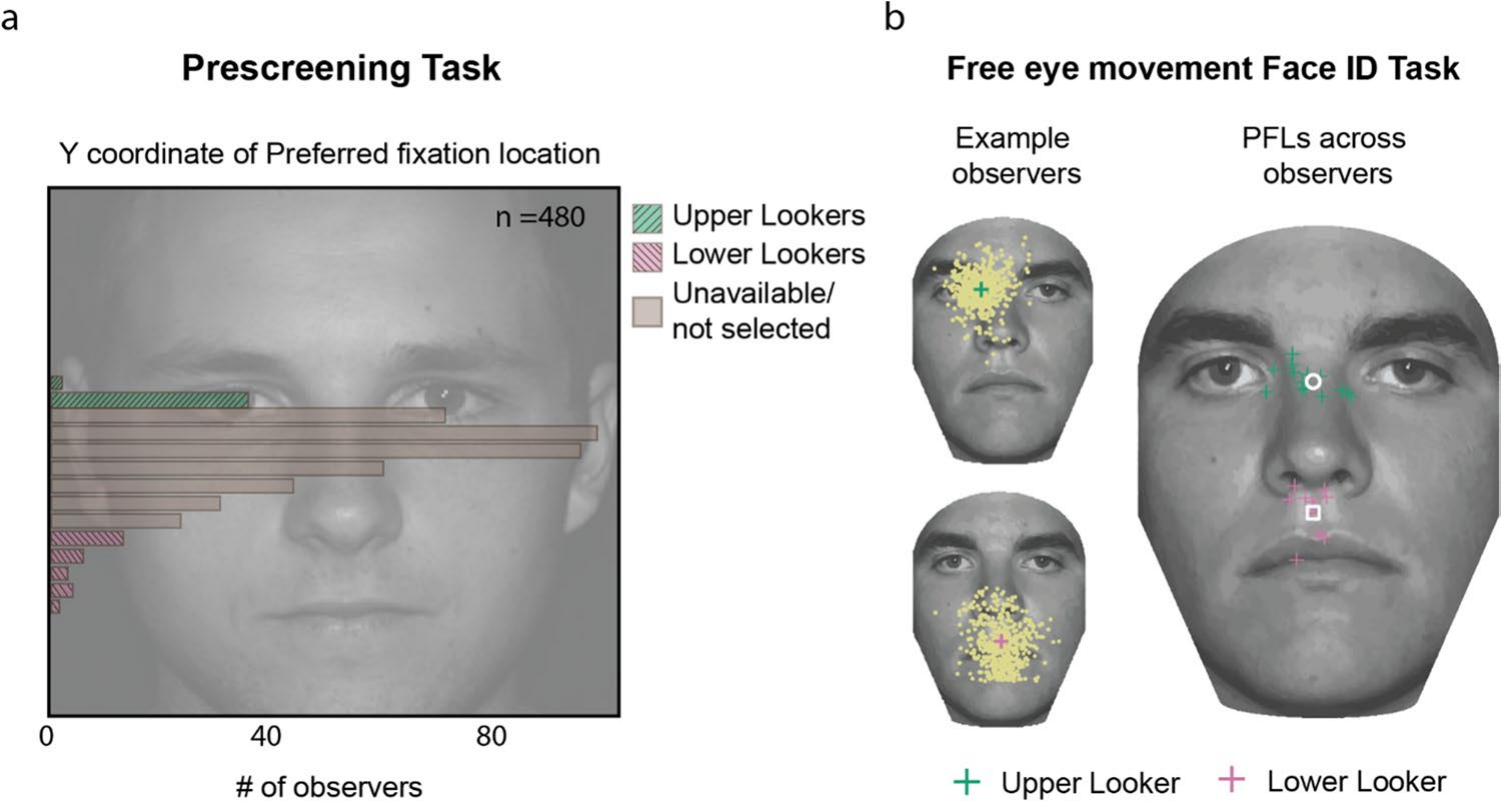
(**a**) Distribution of the vertical coordinates of the preferred fixation positions of 480 observers who participated in the prescreening task. The face in the background serves as a reference to visualize this distribution relative to the facial features. We invited 15 upper and 13 lower lookers from the tails of this sample to participate in the entire study; 14 upper and 11 lower lookers completed the study. (**b**) To verify if the observers we selected maintained their preferred fixation location (PFL) for the faces used in the adaptation experiment, we repeated the free-fixation face identification task using four faces, two of which were the faces used in the adaptation task (see Methods). The left panels show the trial-wise first fixations as yellow dots on the face, an example upper and lower looker, respectively. The PFLs for the example upper and lower looker are depicted by the green and pink crosses, respectively. The panel on the right shows the PFLs of all the upper and lower lookers as green and pink crosses, respectively. The white circle and square indicate the mean PFL of the upper and lower looker groups, respectively

The distribution of the vertical coordinate of the preferred fixation locations (PFL) of the 480 observers in the prescreening task is shown in Fig. 3a. Of these 14 upper lookers and 11 lower lookers completed the entire study. In Fig. 3b, the PFL of participants in these groups are shown as green and pink crosses, respectively.

### Free-eye-movements face identification task

Example first fixation from one upper and one lower looker are shown in the left panel of Fig. 3b. Individual preferred first fixation locations of upper and lower lookers are shown in the panel on the right in green and pink crosses. There was no significant difference in the identification performance across these groups (*t* (23) = 1.71, *p*= 0.1, *μ*_upper_ = 97.81%, *μ*_lower_ = 96.02%). The mean PFLs of the upper and lower looker groups were 4.81% and 61.13% of the eye-mouth distance below the eye level. We selected these two positions on the midline for the subsequent face-matching task.

### Enforced-fixation face-matching task

**Fig. 4:**
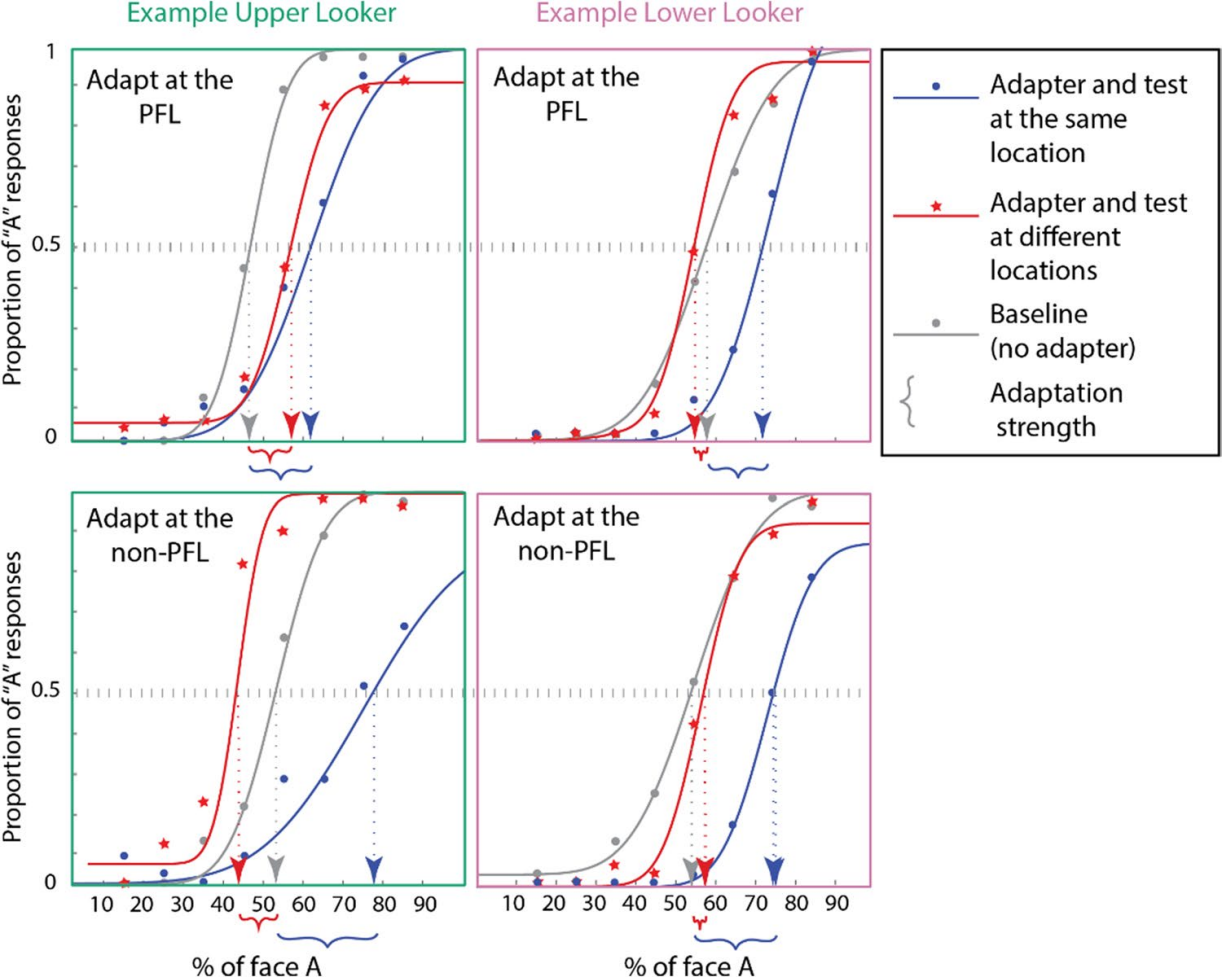
Psychometric fits of one example upper looker and one example lower looker. The panels on the left in green show data from the upper looker, whereas the columns on the right shown in pink show data from the example lower looker. In all panels, the curves in blue and red depict conditions where the adapter and test were at the same or different locations, respectively. The gray curve represents the baseline condition with no adapter. The top panels represent conditions where adaptation happened at the observer's *own group* mean preferred fixation location (PFL; for the upper looker, this was near the eyes, while for the lower looker, this was near the mouth). The lower panels represent conditions where the adaptation happened at the *other group's* mean PFL (for the upper looker, this was near the mouth, while for the lower looker, this was near the eyes). The psychometric fitting was done based on Wichmann & Hill (2001). See Methods for details. The *point of subjective equality* (PSE) is the % of face A for which the observer reports face A and face B with equal probability (50 %). The strength of adaptation for a given condition is calculated as the shift in the PSE relative to the baseline condition

To characterize the strength of the face adaptation effect (FAE), we extracted the point of subjective equality (PSE) for each condition by fitting a psychometric function to the observer's responses to different morph levels. This step is visualized for one upper looker and one lower looker in Fig. 4. Since our goal was to understand if there is a transfer of the FAE across spatial locations, we visualized the conditions where the adapter and test faces were at the same spatial location in blue and the conditions where they were at different spatial locations in red. The baseline condition with no adapter is shown in grey. The point of subjective equality for a given condition can be found by projecting the point where the psychometric curve for that condition has an ordinate of 0.5 (50% probability of responding 'face A') onto the X-axis. The strength of the FAE for a condition was calculated as the shift in the PSE between conditions with and without the adapter, all else being the same.

**Fig. 5:**
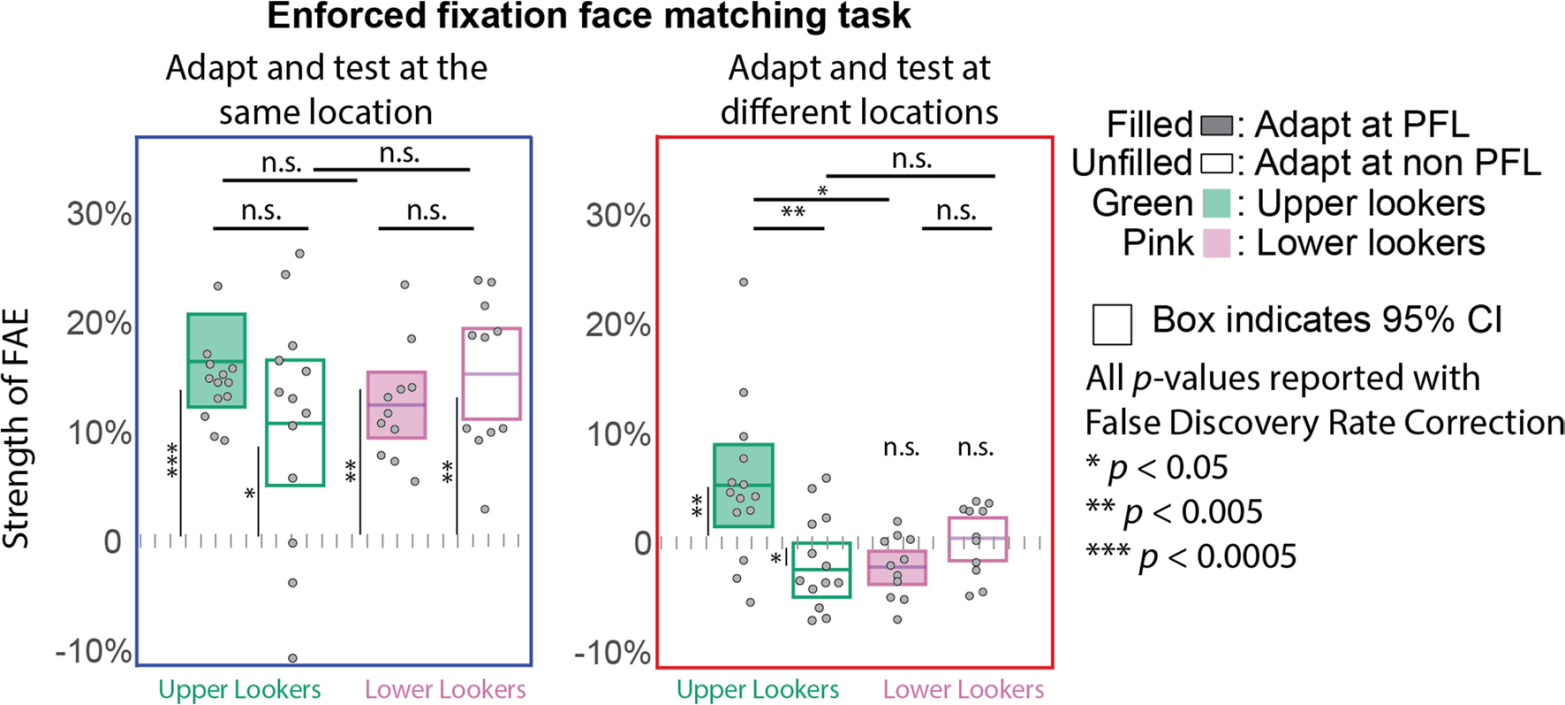
These charts show the pooled adaptation strength across all observers. The left panel (in blue) shows the conditions where the adaptation and test happened at the same spatial location (a high adaptation effect in these conditions would indicate position-specificity of the face-adaptation aftereffect (FAE)). The right panel (in red) shows the conditions where the adaptation and testing happened at different spatial locations (a higher adaptation effect here would indicate position invariance or spatial transfer of the FAE). The green and pink colors indicate upper and lower lookers, respectively. Filled and unfilled boxes indicate conditions where the adaptation happened at the observer's *own group* preferred fixation location (PFL) or the *other group* PFL, respectively

Next, we conducted a three-way mixed factor repeated-measures ANOVA on the strength of the FAE with the *looker type* (upper vs. lower), the *relative position* of the adapter and test (same vs. different), and the *adapter location* relative to the fixation (PFL vs. non-PFL) as factors. There were significant main effects of the *relative position* of the adapter and test (*F* (1, 23) = 77.46, *p* << 0.001, generalized *η*^2^ = 0.488), and a significant interaction effect between *looker type* and *adapter position* relative to the fixation (*F* (1,23) = 18.71, *p* < 0.001, generalized *η*^2^ = 0.064). The mean strengths of the FAEs (defined as the shift in the *point of subjective* equality in the *adaptation* condition relative to the *no adaptation* condition) for different conditions are shown in Fig. 5. The left panel shows that the FAEs were the strongest (and significantly greater than zero) in all conditions where adaptation and testing happened at the same location. There were no significant differences in the strengths of the adaptation effect across all such conditions. The right panel shows the conditions of interest: where adaptation and testing happened at different spatial locations. A significant FAE in these conditions would imply position invariance (or spatial transfer) of the adaptation effect. We conducted three planned contrasts to determine whether upper and lower lookers had any difference in the spatial transfer of the FAE. When adapted at their PFL and tested at their non-PFL, the upper lookers showed a significant transfer of the FAE (*µ =* 4.9%, *t* (23) = 3.28, *p*_*adj*_ = 0.0098, 98.3% CI = [0.0104, 0.087]), but not for the lower lookers (*µ =* -1.3%. *t* (23) = -0.81, *p*_*adj*_ = 1, 98.3% CI = [-0.057, 0.03]). The FAE of the upper lookers when adapted at the PFL and tested at their non-PFL was also significantly lower than the FAE in the condition where they were adapted and tested at their PFL (*µ*_*difference*_* =* 7.1% *t* (23) = 4.408, *p* = 0.0006, 98.3% CI for the difference between means= [0.03, 0.113]). All statistics have been corrected for multiple comparisons using the Bonferroni method for three comparisons. These results suggest a partial spatial transfer of the FAE for the upper lookers when the adaptation happened at their PFL and testing happened at their non-PFL (located 3.6° away). The lower lookers showed no significant spatial transfer of the FAE irrespective of the adaptation location. The results were qualitatively unchanged when the analysis was repeated after excluding outliers (see Methods).

## Discussion

The human face has a non-homogeneous distribution of information, with the eyes and the mouth being the most informative features for everyday face tasks like person and gender identification (Gosselin & Schyns, 2001). Concomitantly, ~ 90% of humans consistently land their first eye movement closer to the eyes in various face tasks (Peterson & Eckstein, 2012), while the rest land their initial eye movement closer to the mouth (Peterson & Eckstein, 2013). Given that humans reach ~ 90% of their peak identification within the first fixation (Hsiao & Cottrell, 2008), and spatially specific coding is increasingly being recognized in higher-order visual processing (Groen et al., 2022), we hypothesized that upper and lower lookers rely on distinct neural representations for face processing. Here, we tested if upper and lower lookers differ in the position-specificity of the face adaptation aftereffect (FAE).

### Do upper and lower lookers have different neural face representations?

We found that the FAE partially transferred to a location 3.6° away from the point of adaptation for upper lookers but not for lower lookers when the adaptation happened at each group’s respective mean PFL. Thus, the upper lookers show some degree of position invariance for the FAE. Further, we found that both upper and lower lookers showed an equally strong FAE when the adaptation and testing happened at the same location (irrespective of the adaptation location). The FAE is known to have position-specific and position-invariant components (Zimmer & Kovács, 2011). Further, Kovács et al. (2008) showed that the position-specific and position-invariant components might be driven by the Occipital Face Area (OFA) and the Fusiform Face Area (FFA), respectively. Based on this framework, we suggest that the upper lookers may rely more on the FFA region than the lower lookers.

Poltoratski et al. (2021) recently measured the position tolerance of face-selective voxels to estimate the population receptive field (pRF) sizes of different face-processing regions for upright and inverted faces. They reported that the pRFs are the largest in the mFUS area (which contains the FFA), ranging from 3° to 5° based on eccentricity from the fixation location. Likewise, for the IOG (which contains the OFA; Grill-Spector et al., 2018), the pRF sizes range from 1.5° to 3° (see Fig. 3; Poltoratski et al., 2021). fMRI voxels pool activity from many neurons with similar response properties. Therefore, larger voxel pRFs suggest larger receptive fields of the underlying neural populations (Dumoulin & Wandell, 2008). Consider two individuals, one relying more on FFA neurons with larger receptive fields versus another relying more on OFA neurons with smaller receptive fields for face processing. After adapting face-selective neurons at one location, testing 3.6° away should engage some of the adapted FFA neurons due to their larger receptive fields of 3–5°. In contrast, the individual relying on smaller OFA neuronal receptive fields (1.5–3°) would activate a distinct unadapted neural population. Thus, we expect a partial transfer of the face adaptation aftereffect only for the individual using FFA neurons more, while the other should show no transfer due to non-overlapping OFA neuronal receptive fields.

What would be the functional purpose of different neural representations between upper and lower lookers? We can get some clues to approach this question from findings in patients with acquired prosopagnosia relating damage to the FFA. Orban de Xivry et al. (2008) report a significantly increased mouth-looking trend in patient *PS* who suffered from prosopagnosia following a head injury that left her with major lesions in her fusiform gyrus. Another study with a larger cohort of 11 acquired prosopagnosia patients also found an increased mouth fixation tendency and related it to damage to the right fusiform face area (Pancaroglu et al., 2016). While the true cause for this shift is difficult to establish experimentally, Caldara et al. (2005) suggest that achieving face recognition using the eye region requires a *simultaneous representation of multiple elements* of the face, while doing so using the mouth region may need *relatively local processing*. As described in Grill-Spector et al. (2017); Poltoratski et al. (2021), the FFA has a larger population receptive field and thus has a greater capacity for spatial integration of information. Along the lines of Caldara et al. (2005), we argue that if the ability to integrate information from multiple elements from a large spatial region were compromised for some reason, it may result in a preference for looking lower on faces. How does this apply to our scenario? We suggest that individual differences in the preference to look lower or higher on the face are developed over time based on various factors that determine the spatial integration ability of each observer.

Our findings are also relevant to the popular theoretical notion that faces are processed holistically: a mode of information processing that considers various features of the face together rather than focusing on parts (Richler et al., 2012). The Composite Face Effect (CFE) is one of the well-known behavioral correlates of holistic face processing (Rossion, 2013). We recently found that upper lookers show a stronger CFE than lower lookers (Chakravarthula & Eckstein, 2022). This finding is consistent with the current line of evidence and suggests that the upper lookers may pool information from a larger region than the lower lookers.

### Does visual experience have a role in the individual differences in the neural face code?

There has been an increasing interest in the question of how spatiotemporal biases in our day-to-day experiences of various visual stimuli shape their neural processing (see Groen et al., 2022, for a discussion). While we can show using the face adaptation aftereffect that upper and lower lookers must have some difference in their neural face codes, we cannot claim that long-term differences in the visual experience of faces owing to differences in oculomotor strategy to view faces *caused* these differences. This is because we used a quasi-experimental manipulation of the PFL rather than experimentally manipulating the lifetime visual experience of faces in a randomized manner. However, we argue that our results are broadly consistent with a growing body of evidence that strengthens the validity of the claim that differences in the long-term visual experience of faces *exist* between upper and lower lookers. Firstly, human PFL is very consistent across a wide range of tasks such as person, emotion, and gender recognition (Peterson & Eckstein, 2012) and even challenging tasks such as ethnicity categorization (Chakravarthula et al., 2021). While this list of tasks is by no means exhaustive, it demonstrates the use of a consistent viewing strategy across a variety of common face-viewing contexts. Secondly, the PFL is remarkably consistent when tested across intervals of up to 2 years (Peterson & Eckstein, 2013). While this finding does not establish the stability of the PFL across much longer timespans, it is a first step in demonstrating the long-term temporal consistency of eye movements to faces. Thirdly, the PFL is preserved in a wide range of viewing contexts – faces in static scenes (Broda & de Haas, 2022a), faces in videos (Broda & de Haas, 2022b) and during real-world interactions (Peterson et al., 2016). These results suggest that our measurements of the PFL in the lab generalize to a wide variety of viewing contexts in and out of the lab. Finally, since faces are the class of visual stimuli humans look the most at (Oruc et al., 2019), small but consistent differences in the first fixation can have a disproportionately higher impact on the visual experience of faces compared to other objects. While these findings support the claim that upper and lower lookers have long-term differences in the visual experience of faces, we cannot make a definitive statement in the absence of a randomized double-blind experimental study.

### What are the implications of our findings for future research?

Kovács et al. (2007) reported that a shorter adaptation time (500 ms) resulted in a position-invariant FAE while a longer adaptation time (~5 s) resulted in a relatively position-specific FAE. Despite using a longer adaptation time of 4 s in our task, the upper lookers showed a transfer of the FAE across spatial locations, suggesting that the adaptation duration and the location of the PFL are independent factors affecting the position-specificity of the FAE. Given some of the contradictory findings regarding the position-specificity of the FAE (see Zimmer & Kovács, 2011), our results emphasize the need to account for individual differences in PFL when designing experiments.

The PFL on the face plays a functional role in a variety of common face tasks such as person, gender, and emotion identification, such that greater proximity of the fixation position to the PFL results in higher task performance (Or et al., 2015; Peterson & Eckstein, 2012). Concomitantly, neural responses to face features are tuned to their position relative to the typical fixation location on the face (Issa & DiCarlo, 2012; Stacchi et al., 2019). Does the position specificity of these neural representations depend on the fixation location relative to the PFL? Our results suggest that the answer depends on the location of the PFL: lower lookers may have a greater degree of position specificity of the neural code relative to the upper lookers.

The result that upper and lower lookers use distinct neural codes for face processing may have important implications for understanding the mechanisms underlying the increased tendency for looking lower on the face in developmental disorders such as autistic spectrum disorder (Tanaka & Sung, 2016), Fragile X syndrome (Hong et al., 2019), and 22q11.2 deletion syndrome (Campbell et al., 2010). Specifically, research relating the pathology of these conditions with changes in neural representations of faces might be useful in developing early diagnostic tools and rehabilitation programs for these conditions.

In summary, idiosyncratic differences in the preferred first fixation location on the face are associated with differences in the position-specificity of the face adaptation aftereffect. Individuals with a preferred fixation location higher up on the face show greater position invariance of the FAE compared to those with a preferred fixation location lower on the face. These differences in position specificity imply differences in underlying neural representations of faces between these two groups.
